# Supplementary material for: Growth Developmental Defects of Mitochondrial Iron Transporter 1 and 2 Mutants in Arabidopsis in Iron Sufficient Conditions
Source: Plants (Basel). 2023 Mar 4;12(5):1176. doi: 10.3390/plants12051176 (PMC10007191; doi:10.3390/plants12051176)
Supplement: Supplementary file 1 [file plants-12-01176-s001.zip › Supplementary Table S1.pdf]

Supplementary Table S1. List of primers used in this work

Primer	used for	Orientation	Primer (5'-3')
1	Amplification of *ADH1* promoter	Forward	AGCGGATAACAATTTCACACAGGAAACAGCTAGGCGCATGCAACTTCTTTTC
2	Amplification of *ADH1* promoter to be fused to *MIT2* cds	Reverse	TTTGGGTACGGTTGTAGCCTCCGTAGCCATCATATGTATATGAGATAGTTGATTGTATGCTTGGTATAG
3	Amplification of *ADH1* promoter to be fused to *MIT1* cds	Reverse	TGGGAATTTGGTTGTTGCTTCTGTTGCCATCATATGTATATGAGATAGTTGATTGTATGCTTGGTATAG
4	Amplification of *MIT2* cds	Forward	ATGGCTACGGAGGCTACAAC
5	Amplification of *MIT2* cds	Reverse	TCAGGCAGTGTTTGAATCGAC
6	Amplification of *MIT1* cds	Forward	ATGGCAACAGAAGCAACAACCA
7	Amplification of *MIT1* cds	Reverse	TCAAGCTGCGTTTGCTTCACCAT
8	Amplification of *ADH2* terminator to be fused to *MIT2* cds	Forward	GACTTCAATGTCGATTCAAACACTGCCTGATTTGTAATACGACTCACTATAG
9	Amplification of *ADH2* terminator	Reverse	GGTAACGCCAGGGTTTTCCCAGTCACGACGGCCGGTAGAGGTGTGGTCAATAAG
10	Amplification of *ADH2* terminator to be fused to *MIT1* cds	Forward	GATCTCAATGGTGAAGCAAACGCAGCTTGATTTGTAATACGACTCACTATAG
11	Genotyping of *mit2* mutants and *MIT2* expression by RT-PCR	Forward	TTGCAGATGGGTGAAGGGAC
12	Genotyping of *mit2* mutants and *MIT2* expression by RT-PCR	Reverse	GCAGGATCCTCAAGAGTGTGA
LBb1.3	Genotyping of SALK mutants	-	ATTTTGCCGATTTCGGAAC
LB1sail	Genotyping of *mit2-2* mutant	-	GCCTTTTCAGAAATGGATAAATAGCCTTGCTTCC
RB	Characterization of T-DNA insertion in *mit2-1*	-	TGGAACGTCAGTGGAGCATT
13	Genotyping of *mit1* mutants and *MIT1* expression by RT-PCR	Forward	GTCATTTCTCCAATCTTCTCGG
14	Genotyping of *mit1* mutants and *MIT1* expression by RT-PCR	Reverse	CTGTTTCATAAGTGGACCAGC
15	Genotyping of *mit1* mutants and *MIT1* expression by RT-PCR	Forward	TCTCCAATCTTCTCGGAACCC
16	Genotyping of *mit1* mutants and *MIT1* expression by RT-PCR	Reverse	AGCATGCTCAGGCAACATCT
17	*MIT2* expression by RT-PCR	Forward	AGATAATGTAGTCCACCACCACAC
18	*MIT2* expression by RT-PCR	Reverse	AAGAAAAGCAGGATCCTCAAGAGT
19	*MIT2* expression by RT-qPCR	Forward	TGCAATGTCAGGGTGTGTGT
20	*MIT2* expression by RT-qPCR	Forward	ATGTCAGGGTGTGTGTGGAT
21	*MIT2* expression by RT-qPCR	Reverse	CGGGAGCCATCCCCTTAGAA
At4g24550F	Clathrin adaptor expression by RT-qPCR	Forward	AATACGCGCTGAGTTCCCTT
At4g24550R	Clathrin adaptor expression by RT-qPCR	Reverse	AGCACCGGGTTCTAACTCAA
At4g34270F	TIP41-like expression by RT-qPCR	Forward	GCCAAGCTCATGGTTCCTCC

At4g34270R	TIP41-like expression by RT-qPCR	Reverse	TGCCTCATCTTCGCCAAACC
At2g21640F	UPOX expression by RT-qPCR	Forward	AATCGAAACCGAGAACCCGC
At2g21640R	UPOX expression by RT-qPCR	Reverse	GGTTTGCAAAGAAAGTGGCGT
At5g09570F	MSM1 expression by RT-qPCR	Forward	CTCAGCCTAGTAGCGGTGGT
At5g09570R	MSM1 expression by RT-qPCR	Reverse	GCAGTTCCAGTACCCCAAGC
At3g22370F	*AOX1A* expression by RT-qPCR	Forward	GGAATCGCGAGCTATTGGGG
At3g22370R	*AOX1A* expression by RT-qPCR	Reverse	ACGTTTCCCATGGCCTGAAA
At4g19690F	*IRT1* expression by RT-qPCR	Forward	CAAATGCACAGCTCTTGCGA
At4g19690R	*IRT1* expression by RT-qPCR	Reverse	AATCCAATGACCACCGAGTGAA
At1g01580F	*FRO2* expression by RT-qPCR	Forward	GGTTATGGTGTGCGGAGGAA
At1g01580R	*FRO2* expression by RT-qPCR	Reverse	TTTCCTTTTGGCTTGTGGCG

Start and stop codons are underlined
