# Supplementary material for: Growth Developmental Defects of Mitochondrial Iron Transporter 1 and 2 Mutants in Arabidopsis in Iron Sufficient Conditions
Source: Plants (Basel). 2023 Mar 4;12(5):1176. doi: 10.3390/plants12051176 (PMC10007191; doi:10.3390/plants12051176)
Supplement: Supplementary file 1 [file plants-12-01176-s001.zip › Supplementary Table S2.pdf]

Supplementary Table S2. Genotype of F2 plants from the cross *mit1-1* x *mit2-1*

Plant genotype	Nº of F2 plants	Expected (Mendelian inheritance)
MIT1MIT1 MIT2MIT2	10	11 (1/16)
MIT1mit1 MIT2MIT2	36	22 (2/16)
MIT1MIT1 MIT2mit2	16	22 (2/16)
MIT1MIT1 mit2mit2	9	11 (1/16)
mit1mit1 MIT2MIT2	7	11 (1/16)
MIT1mit1 MIT2mit2	73	44 (4/16)
mit1mit1 MIT2mit2	4	22 (2/16)
MIT1mit1 mit2mit2	22	22 (2/16)
mit1mit1 mit2mit2	0	11 (1/16)
Total Nº of F2 plants analyzed	177	

Homozygous *mit1-1* mutant plants (female parent) were crossed with homozygous *mit2-1* mutant plants (male parent). Seeds of three selfed F1 double heterozygous plants (*MIT1mit1 MIT2mit2*) were directly sown on soil and one hundred and seventy seven F2 plants were genotyped as described in Methods. Expected numbers according to Mendelian inheritance are shown in the last column.
