# Supplementary figures and images for: Growth Developmental Defects of Mitochondrial Iron Transporter 1 and 2 Mutants in Arabidopsis in Iron Sufficient Conditions
Source: Plants (Basel). 2023 Mar 4;12(5):1176. doi: 10.3390/plants12051176 (PMC10007191; doi:10.3390/plants12051176)

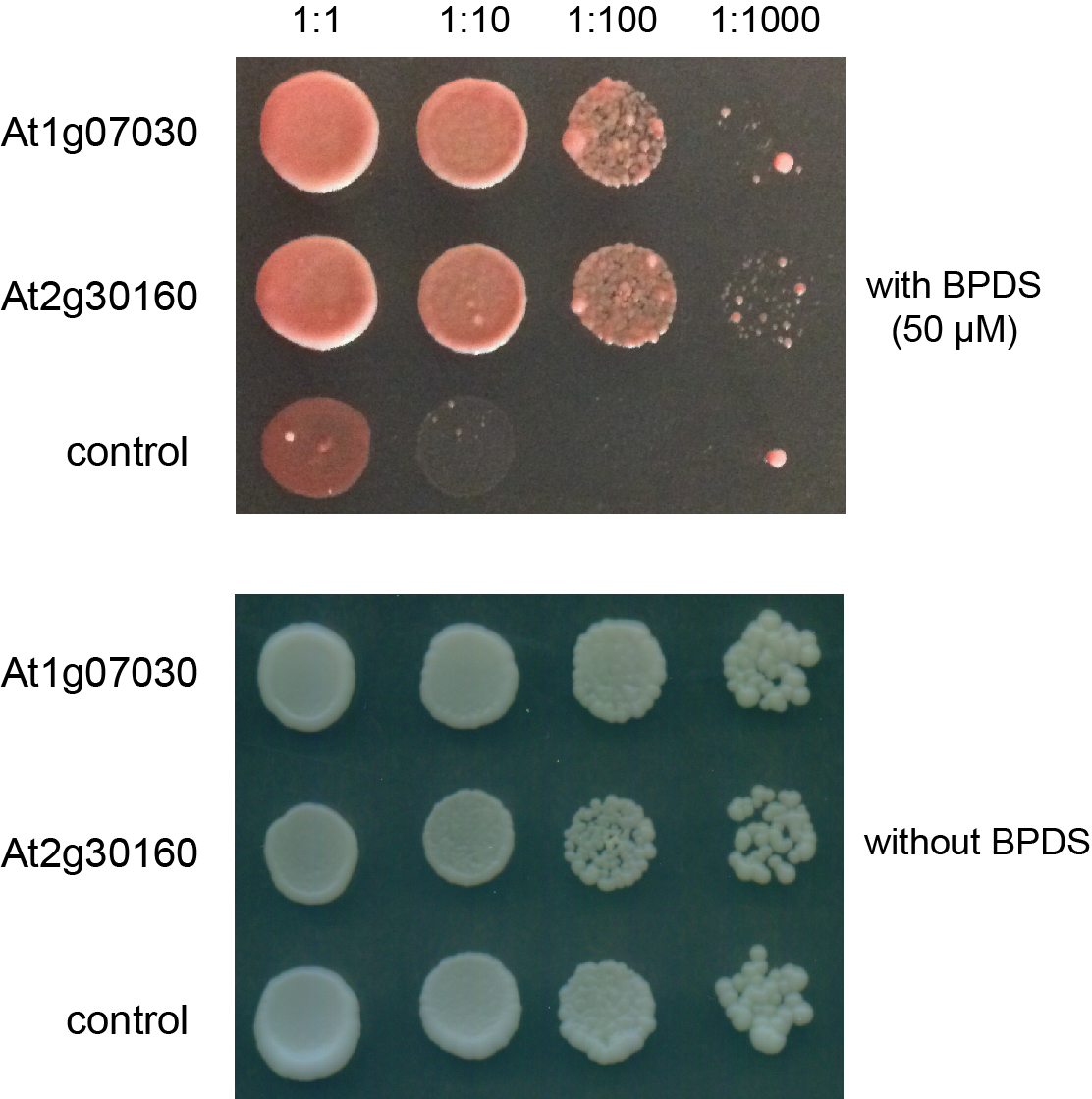

Supplement: Supplementary file 1 [file plants-12-01176-s001.zip › Supplementary Figure S1 working.jpg]

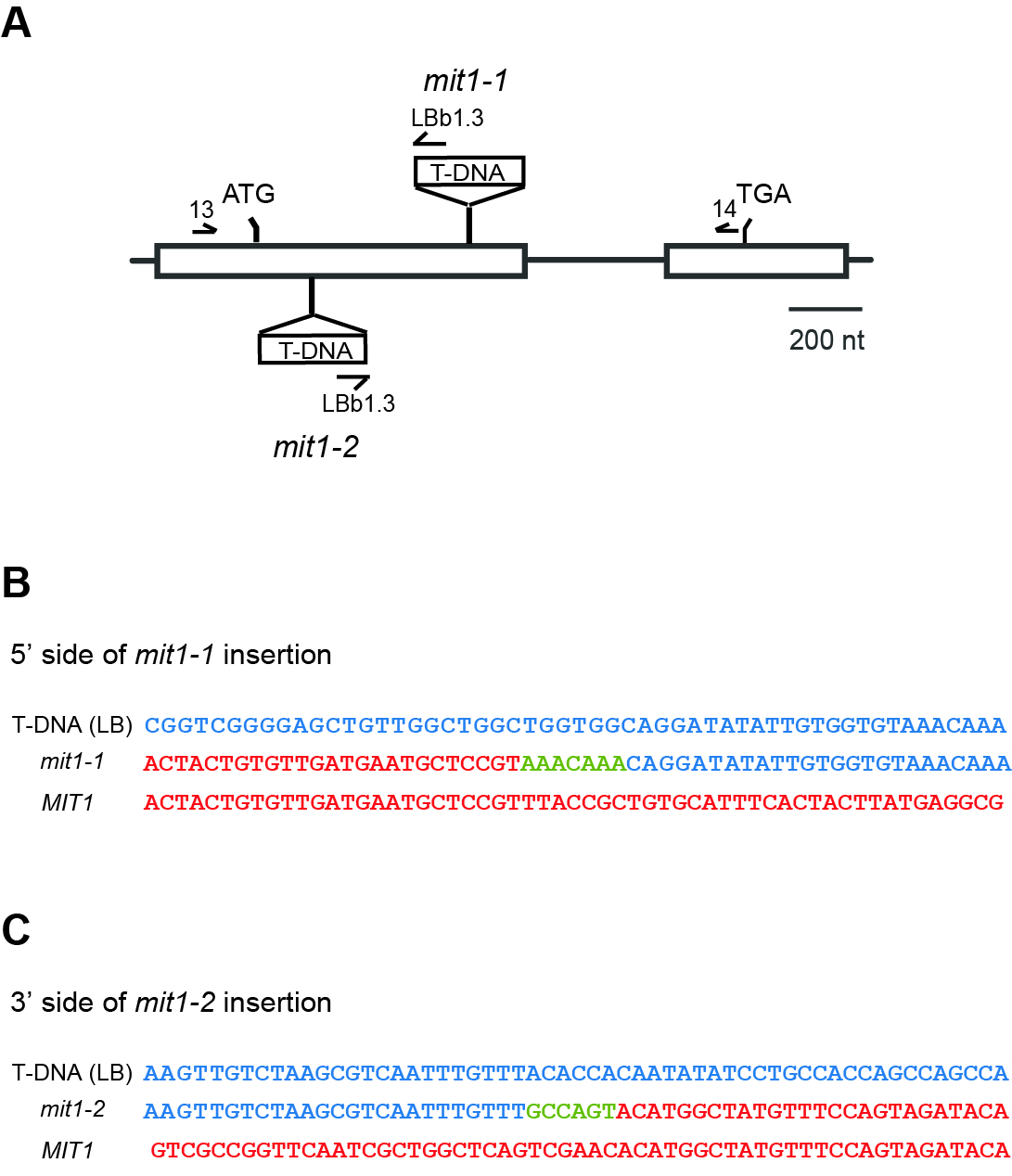

Supplement: Supplementary file 1 [file plants-12-01176-s001.zip › Supplementary Figure S2 working.jpg]

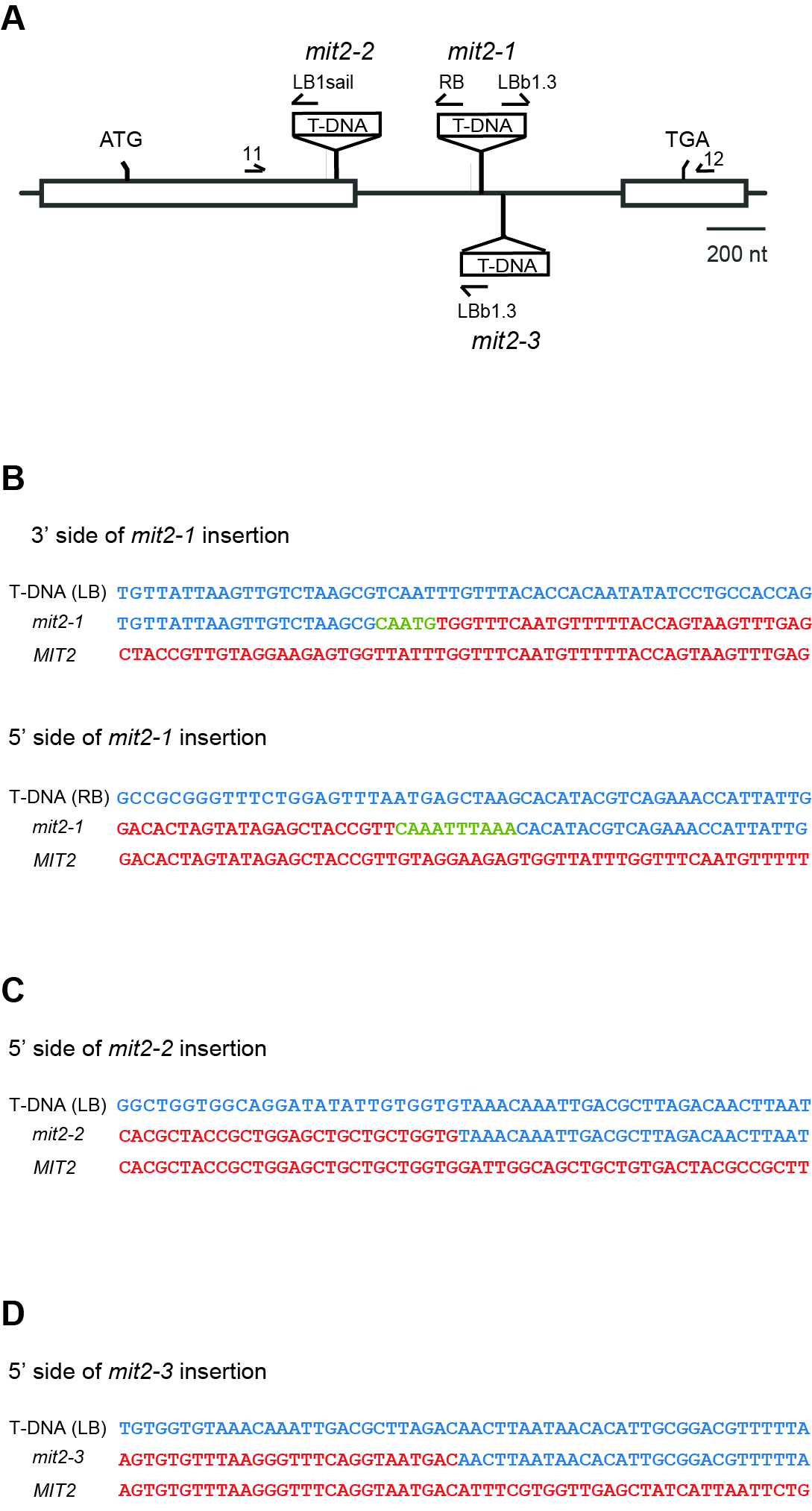

Supplement: Supplementary file 1 [file plants-12-01176-s001.zip › Supplementary Figure S3 working.jpg]

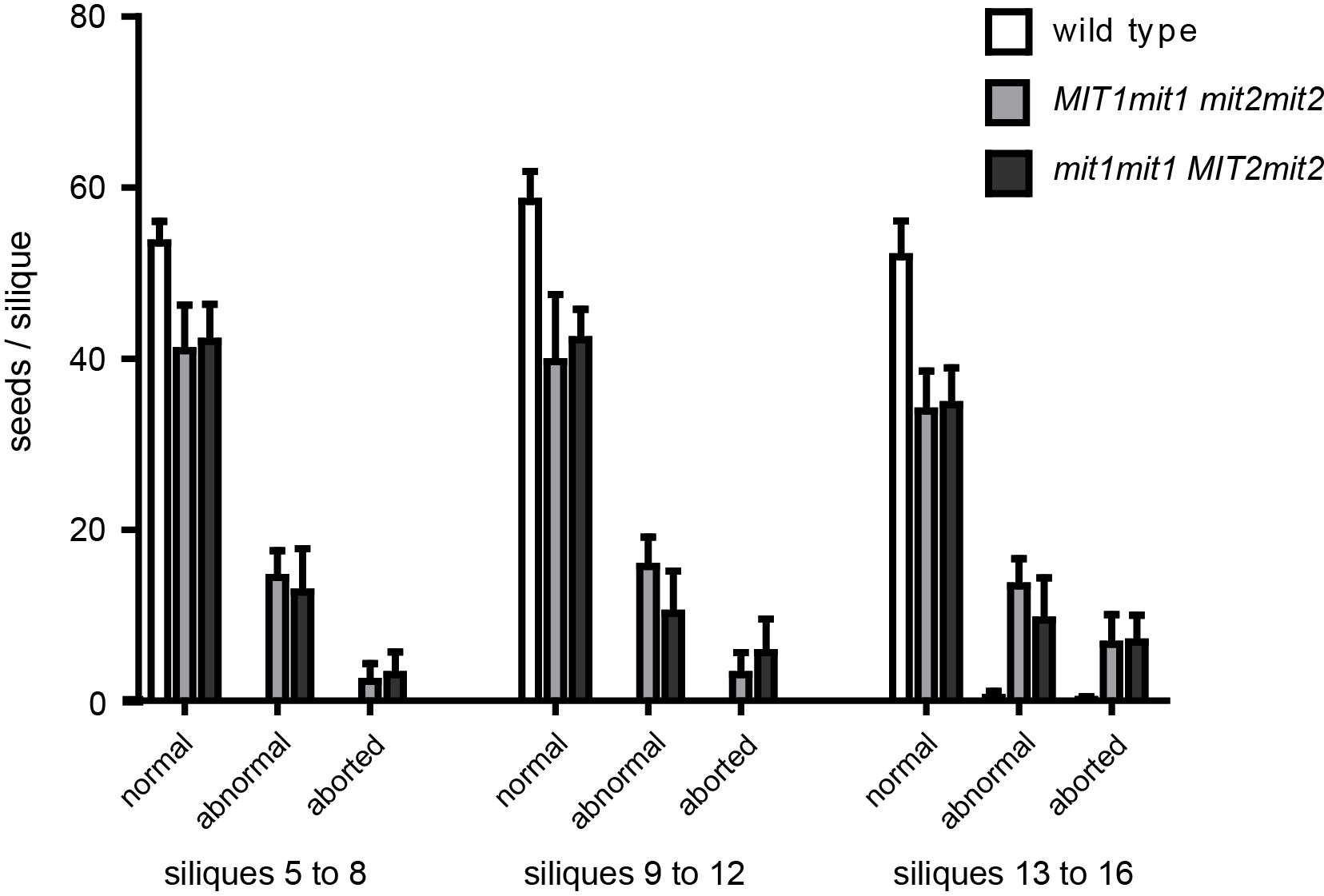

Supplement: Supplementary file 1 [file plants-12-01176-s001.zip › supplementary figure S4 working.jpg]

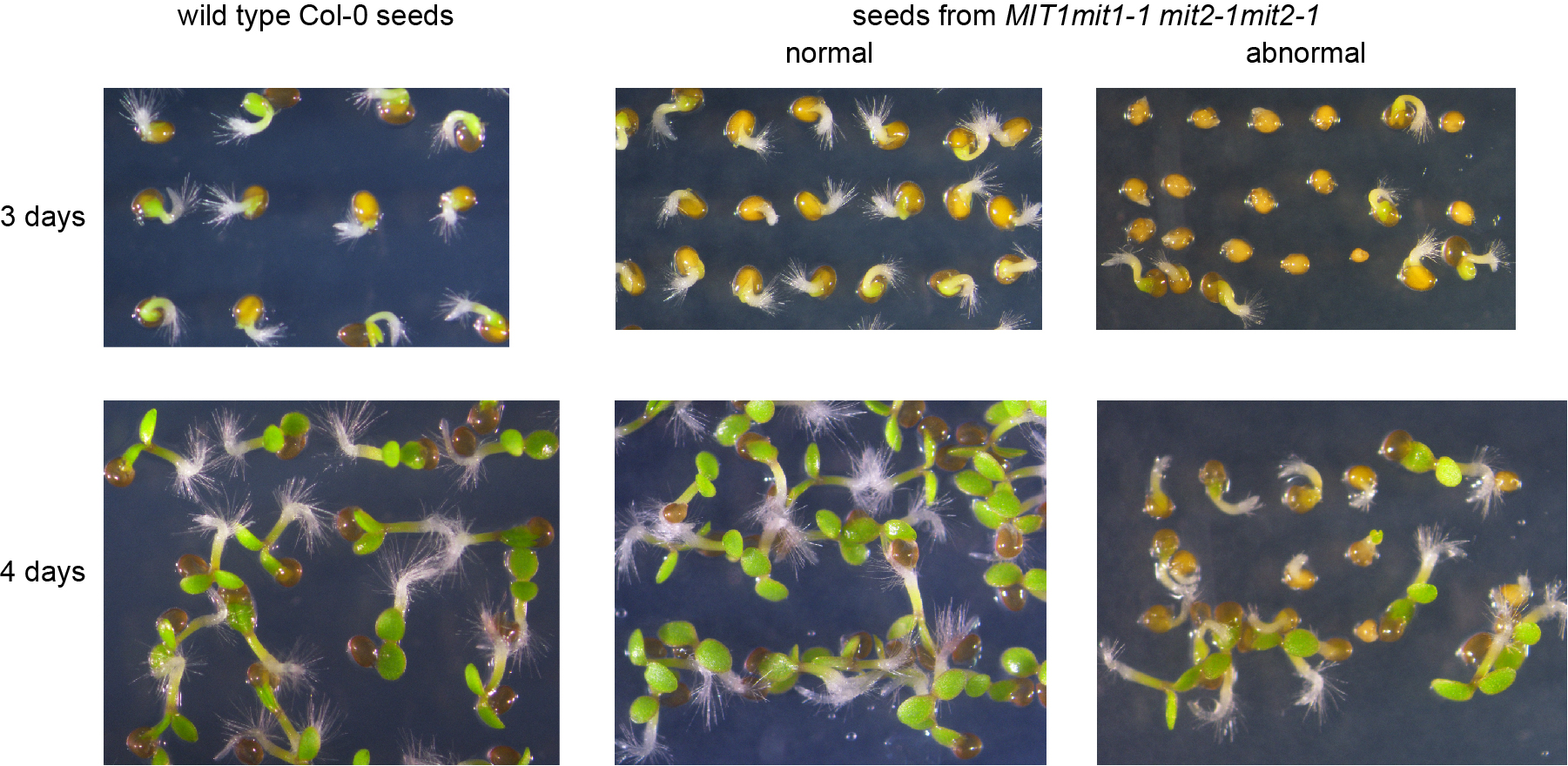

Supplement: Supplementary file 1 [file plants-12-01176-s001.zip › Supplementary Figure S5 working.jpg]

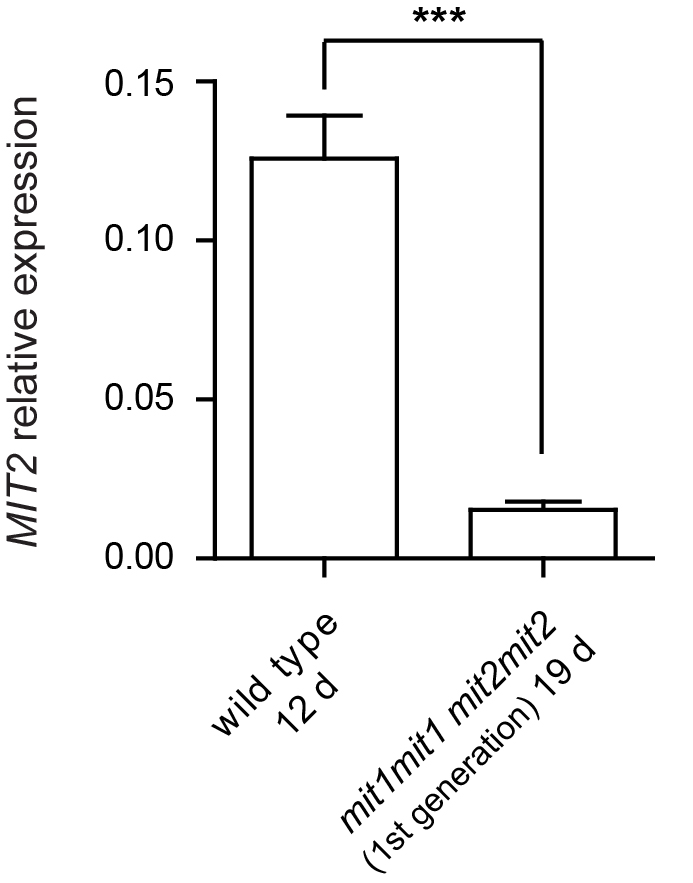

Supplement: Supplementary file 1 [file plants-12-01176-s001.zip › Supplementary Figure S6 working.jpg]

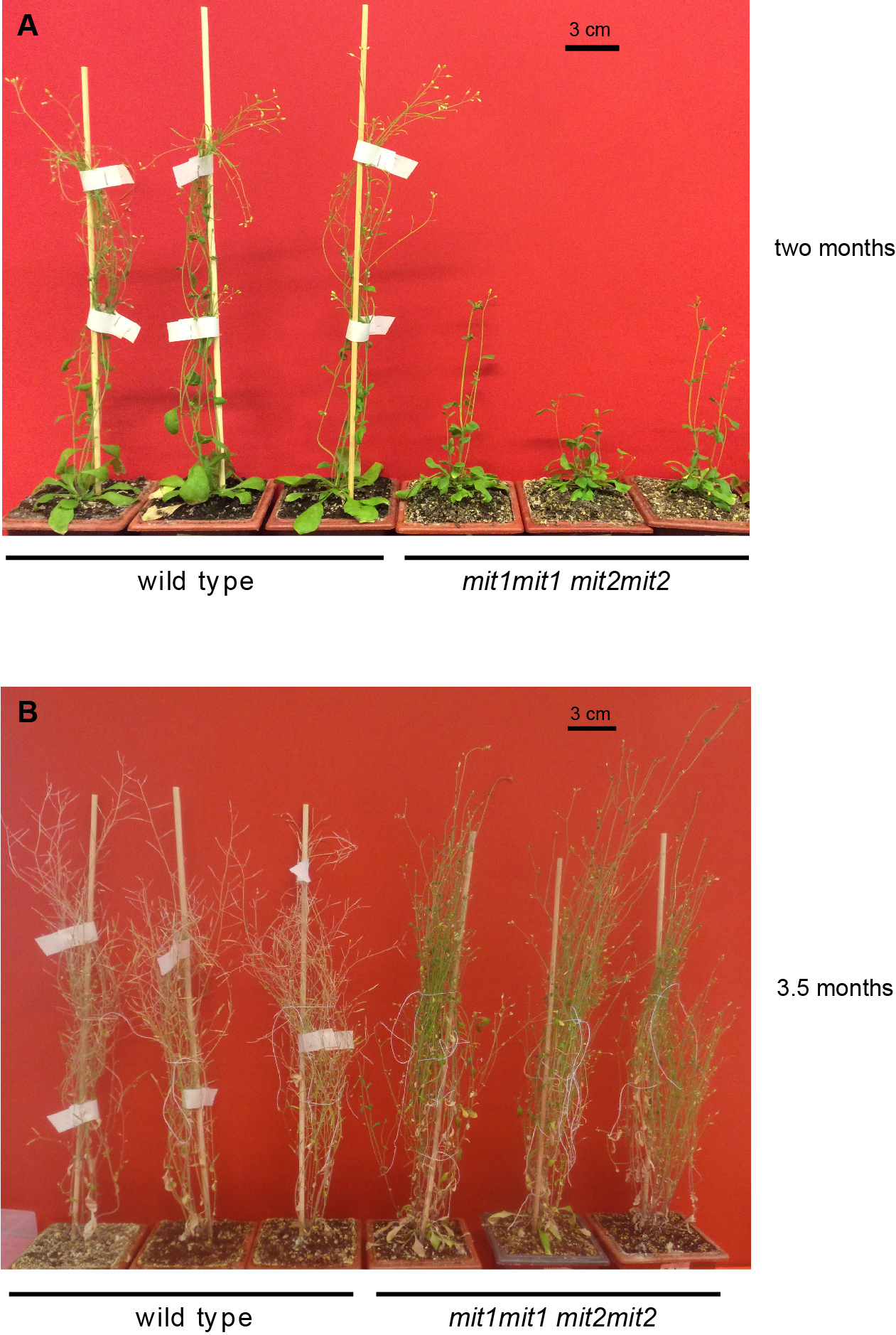

Supplement: Supplementary file 1 [file plants-12-01176-s001.zip › Supplementary Figure S7 working.jpg]

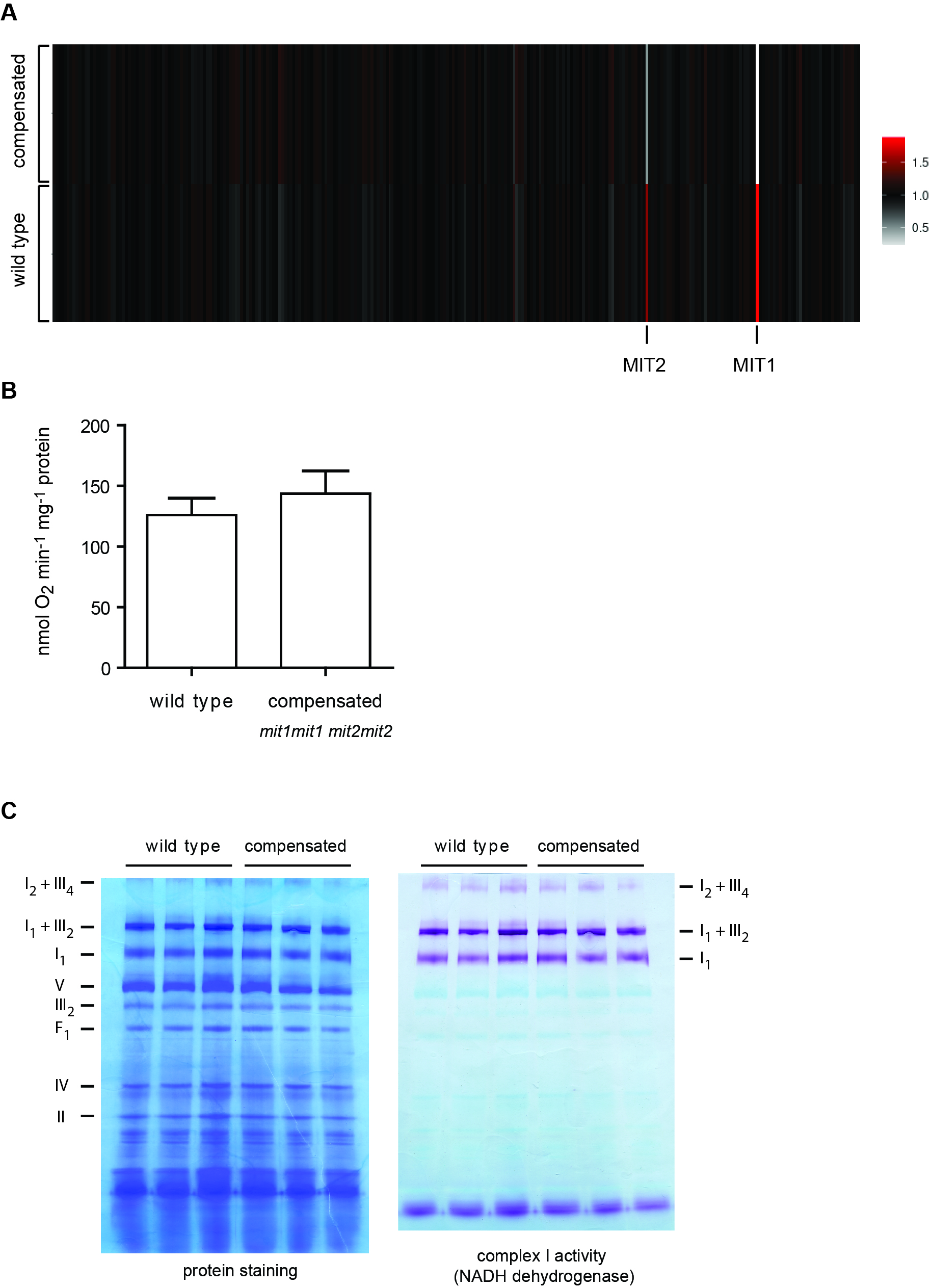

Supplement: Supplementary file 1 [file plants-12-01176-s001.zip › Supplementary Figure S8 working.jpg]
